# Glutamine Supplementation Preserves Glutamatergic Neuronal Activity in the Infralimbic Cortex, Which Delays the Onset of Mild Cognitive Impairment in 3×Tg-AD Female Mice

**DOI:** 10.3390/nu15122794

**Published:** 2023-06-19

**Authors:** Ji Hyeong Baek, Jae Soon Kang, Miyoung Song, Dong Kun Lee, Hyun Joon Kim

**Affiliations:** 1 Department of Anatomy and Convergence Medical Sciences, Institute of Health Sciences, Tyrosine Peptide Multiuse Research Group, Anti-Aging Bio Cell Factory Regional Leading Research Center, Gyeongsang National University Medical School, 15 Jinju-daero 816 Beongil, Jinju 52727, Gyeongnam, Republic of Korea; 2 Department of Physiology, Institute of Health Sciences, Gyeongsang National University Medical School, 15 Jinju-daero 816 Beongil, Jinju 52727, Gyeongnam, Republic of Korea

**Keywords:** mild cognitive impairment, 3×Tg-AD, glutamine, glutamatergic neuronal activity, oxidative stress

## Abstract

It was recently found that glutamine (Gln) supplementation activates glutamatergic neurotransmission and prevents chronic-stress-induced mild cognitive impairment (MCI). In this study, we evaluated the effects of Gln on glutamatergic activity in the medial prefrontal cortex and the onset of cognitive impairment in a triple-transgenic Alzheimer’s disease mouse model (3×Tg-AD). Female 3×Tg-AD mice were fed a normal diet (3×Tg) or a Gln-supplemented diet (3×Tg+Gln) from 2 to 6 months of age. Glutamatergic neuronal activity was analyzed at 6 months, and cognitive function was examined at 2, 4, and 6 months. 3×Tg mice exhibited a decrease in glutamatergic neurotransmission in the infralimbic cortex, but 3×Tg+Gln mice did not. The 3×Tg group showed MCI at 6 months of age, but the 3×Tg+Gln group did not. The expressions of amyloid peptide, inducible nitric oxide synthase, and IBA-1 were not elevated in the infralimbic cortex in the 3×Tg+Gln group. Therefore, a Gln-supplemented diet could delay the onset of MCI even in a mouse model predisposed to cognitive impairment and dementia through genetic modification.

## 1. Introduction

Mild cognitive impairment (MCI) is a stage between normal age-related memory decline and dementia. It induces difficulties in planning, memory, and decision making, which are not sufficient to affect daily activities [1]. MCI patients show an increased incidence of dementia due to neurological disorders, including Alzheimer’s disease (AD) [2,3]. Therefore, the importance of early treatment at the MCI stage before it progresses completely to dementia is increasingly emphasized. However, there is currently no standardized test for MCI due to the complexity of risk factors and low clarity of testing. Thus, there is no specific accepted treatment or drug for MCI [1]. Developing a safe and easily accessible MCI treatment, which is applicable even to ambiguous MCI, will greatly assist in the prevention and early treatment of MCI.

Previous studies have shown that hypoactive glutamatergic neurotransmission in the prefrontal cortex (PFC) is a major pathological feature of depression and cognitive impairments [4,5,6]. The PFC is essential in executive functions and affects cognition and psychopathology. Glutamatergic neurons are densely distributed in the PFC. Glutamatergic neurotransmission is predominantly regulated by tripartite synapses composed of pre- and post-synaptic neurons and glial cells, including astrocytes and microglia [7]. Glutamine synthetase (GS) activity and glutamate–glutamine (Glu-Gln) homeostasis in the PFC are important for glutamatergic activity. Furthermore, Gln plays a pivotal role in astrocyte–neuron interactions in glutamatergic signaling [4,5,6].

Since Gln is a precursor of a crucial excitatory neurotransmitter, Glu, normal neurotransmission can only be maintained when the extracellular and intracellular levels of Gln, along with Glu, are regulated within a specific range [7,8]. However, the transport and recycling of Glu and Gln via the Glu-Gln cycle are susceptible to stress, glucocorticoids, and oxidative/nitrative stress [6,7,9]. Alterations in the Glu-Gln cycle and Glu-Gln levels in the brain lead to impaired glutamatergic neurotransmission and cognitive impairment [9,10,11,12,13]. In mice, decreased GS activity during postnatal synaptogenesis results in spatial memory impairment that persists into adulthood [12]. Therefore, preserving the integrity of the Glu-Gln cycle through supplementation or reducing detrimental stressful factors would be an effective strategy to protect glutamatergic neuronal activity and cognitive functions.

However, since Glu itself is a neurotransmitter, the direct application of Glu can cause neurotoxicity. Since Gln does not induce neurotoxicity like Glu and can be used for energy metabolism, it has been studied as a glutamatergic neuroprotective agent [5,6,9,14,15]. Previous studies have demonstrated that a Gln-supplemented diet and direct infusion of Gln into the PFC activate glutamatergic signaling, increase Glu-Gln levels in the PFC, normalize blood levels of the stress hormone corticosterone, and regulate the expression levels of Gln-Gln transporters in the PFC and hippocampus. Ultimately, these interventions improve chronic immobilization stress (CIS)-induced depression and cognitive impairment in mouse models [6,9,14,15].

Moreover, another mechanism underlying the neuroprotective function of Gln is the regulation of oxidative stress and inflammatory responses. Constant or repetitive increases in reactive oxygen/nitrogen species (ROS/RNS) contribute to chronic oxidative stress and inflammation leading to neuronal damage and disorders such as MCI and AD [16,17,18,19]. A previous study demonstrated that Gln supplementation improved CIS-induced MCI in mice by reducing ROS/RNS in the PFC, hippocampus, and plasma. Gln decreased the expression levels of ROS/RNS-producing enzymes, including inducible nitric oxide synthase (iNOS) and NADPH oxidases (NOX), while increasing synaptic density in the PFC and hippocampus [14].

Previous studies have suggested that Gln holds promise as a candidate protective agent preserving glutamatergic activity and cognitive function. However, the efficacy and mechanism of Gln have thus far been evaluated only in mouse models of CIS-induced depression and MCI, without considering genetic models for dementia. Consequently, the effectiveness of Gln in addressing dementia caused by amyloid beta (Aβ) and tau has been limited. To further investigate the potential of Gln as a candidate for inhibiting MCI, this study aimed to examine the effects of a Gln-supplemented diet on the pathogenesis of MCI in the widely used 3×Tg-AD mouse model, which is commonly employed in MCI and AD research. The 3×Tg-AD triple-transgenic mouse model harbors *APP*_Swe_, *PS1*_M146V_, and *tau*_P301L_, which results in amyloid deposits in the brain and memory deficits that typically manifest at approximately 4–6 months of age. By 12–15 months, extensive Aβ deposits and plaque formation, as well as the presence of hyperphosphorylated tau, are observed in the hippocampus [20,21,22]. Aβ-induced oxidative stress and glutamatergic dysfunction may be the main contributors to MCI in 3×Tg-AD mice [23,24]. Given that Gln activates glutamatergic signaling and inhibits ROS/RNS induction, it is hypothesized that Gln could hinder the onset of MCI in 3×Tg-AD mice [14]. Thus, this study employed a 3×Tg-AD mouse model to explore and solidify the effects of a Gln diet on glutamatergic activity and cognitive function.

## 2. Materials and Methods

### 2.1. Animals

A breeding pair of homozygous 3×Tg-AD [B6;129-Tg(*APP*_Swe_,*tau*_P301L_)1Lfa *Psen1^TM1Mpm^*/Mmjax] mice was provided by the laboratory of Prof. Dong-Gyu Jo at Sungkyunkwan University and bred in a specific pathogen-free animal facility at the Gyeongsang National University Medical School. The room temperature was maintained at a constant level of 25 ± 1 °C, and a 12 h light/dark cycle was followed, with lights turning on at 06:00. The mice were group-housed, with 4–5 mice per cage, and they had free access to water and rodent chow. The mice were bred at 8–16 weeks of age, periodically genotyped to maintain their homogeneity, and outbred with C57BL/6 mice to reduce the risk of deleterious recessive genes being expressed. Body weight, food intake, and behavior were continuously observed to confirm the health status of the mice. Female wild-type (WT, C57BL/6) and 3×Tg-AD mice were fed either AIN-93G (normal diet) or a Gln-supplemented diet (150 mg Gln/kg AIN-93G) (Raonbio, Yongin, Republic of Korea). The 3×Tg-Vglut2-ires-Cre::tdTomato strain was generated by crossing 3×Tg-AD mice with Vglut2-ires-Cre::tdTomato mice [6], resulting in mice with fluorescently labeled glutamatergic neurons. At 2 months of age, the mice were divided into 2 groups and fed a normal diet or a Gln-supplemented diet. Glutamatergic neurotransmission activity was investigated at 6 mon. All animal experiments were conducted in accordance with the National Institutes of Health (NIH) guidelines. The protocol was approved by the Gyeongsang National University Institution Animal Care and Use Committee (GNU-190819-M0039).

### 2.2. Glutamatergic Neurotransmission Activity Analysis

Spontaneous excitatory postsynaptic currents (sEPSCs) from glutamatergic neurons were recorded in the IL of 6-month-old female mice. Brains were sectioned transversely, and sEPSCs were recorded as previously described [4,6]. Fluorescence-labeled glutamatergic neurons in the IL were visualized using an infrared-differential interference contrast microscope (Olympus BX51WI, Olympus Optical, Tokyo, Japan). The recording chamber was continuously perfused with artificial cerebrospinal fluid at a rate of 1.5–2 mL per min and kept at 30 ± 2 °C. The cell was held at −70 mV for whole-cell voltage-clamp recording, and the sEPSC was recorded for a duration of over 10 min. The following reagents were used to prepare the pipette electrolyte: 130 mM KCl, 5 mM CaCl_2_, 10 mM HEPES, 10 mM EGTA, 0.5 mM Na_2_GTP, 2 mM MgATP, and 5 mM phosphocreatine. Picrotoxin (100 μM) was used to isolate the glutamatergic current by inhibiting GABA receptors. Signals were digitized with a MultiClamp 700B. sEPSC peaks were detected and analyzed using Clampfit (Molecular Devices, RRID:SCR_011323), Mini Analysis (Synaptosoft, RRID:SCR_002184), and OriginPro (OriginLab, RRID:SCR_014212). From the 10 min data, stable sEPSC signals with a duration of 2 min were selected for further analysis.

### 2.3. Object Recognition Test (ORT)

To assess recognition memory, the mice underwent training and testing as previously described [14]. The experiments were conducted between 18:30 and 22:00 during the dark phase under 50-lux light conditions. The ORT was performed in an acrylic box (33 × 33 × 20 cm) with 1 cm of bedding, following 3 steps. (1) Habituation: Each mouse was transported to the box and allowed to freely explore for 10 min/day for 2 days. (2) Training: On day 3, each mouse was exposed to 2 identical objects in the box for 10 min. The mouse was positioned in the box with its nose facing away from the objects. (3) Test: After a 24 h interval, 1 of the objects was replaced with a novel object, and each mouse was given 5 min to explore. The animal behaviors were recorded, and the object exploration time was automatically analyzed using EthoVision software (Noldus Information Technology, Wageningen, The Netherlands). A mouse’s nose approaching within 1 cm of the object was defined as ‘exploration’. The discrimination index (DI) for the ORT was calculated as follows:

$$\mathrm{DI}=\frac{\mathrm{Difference}\;\mathrm{in}\;\mathrm{exploration}\;\mathrm{duration}\;(\mathrm{novel}-\mathrm{familiar})}{\mathrm{Total}\;\mathrm{exploration}\;\mathrm{duration}\;(\mathrm{novel}+\mathrm{familiar})}$$

The ORT was conducted with 2-, 4-, and 6-month-old 3×Tg-AD and WT female mice.

### 2.4. Total ROS/RNS Assay

The mice were decapitated at 6 months of age, and trunk blood was collected in a K_3_EDTA-coated vacutainer at 09:30–10:30 a.m. The blood was centrifuged at 1000× *g* for 15 min at 4 °C to isolate plasma. Plasma was stored at −80 °C until use. Total plasma ROS/RNS were analyzed using an OxiSelect In Vitro ROS/RNS Assay Kit (Cell Biolabs, San Diego, CA, USA). Briefly, plasma was incubated with a catalyst that accelerates the oxidative reaction. A 2′,7′-dichlorodihydrofluorescein (DCFH) probe was added, and the oxidation reaction proceeded. Fluorescence levels in the samples were measured against DCF standards using a microplate reader (Victor Nivo, Perkin Elmer, Waltham, MA, USA). Each sample was evaluated in duplicate.

### 2.5. Neuron-Derived Exosome (NDE) Isolation and Aβ_1–42_ Enzyme-Linked Immunosorbent Assay (ELISA)

According to a previous study [25], NDEs were isolated from plasma with some modifications. Exosomes were collected using the Total Exosome Isolation Reagent from Plasma (Invitrogen, Carlsbad, CA, USA) and resuspended in PBS (pH 7.4) with protease and phosphatase inhibitors. The exosome suspensions were incubated with the anti-mouse L1CAM-biotinylated antibody (CD171, LS-C685295, LSBio, Seattle, WA, USA, 1:200) overnight at 4 °C and bound to Dynabeads M-280 Streptavidin (Invitrogen) for 2 h at 4 °C. NDEs were eluted using 50 mM glycine-HCl (pH 3.0), and 1 M Tris-HCl (pH 8.0) was added to neutralize the solution. NDE proteins were extracted using M-PER (Thermo Fisher Scientific, Waltham, MA, USA), containing 0.15% bovine serum albumin and protease and phosphatase inhibitors. The samples were frozen and thawed twice, vortexed, and centrifuged for 3 min at 12,000× *g* and 4 °C. Aβ_1–42_ levels in the NDEs were analyzed using ELISA (KMB3441, Invitrogen) and normalized with the tetraspanin exosome marker CD81 (CSB-EL004960MO, Cusabio, Houston, TX, USA).

### 2.6. Immunohistochemistry (IHC)

The mice were anesthetized with avertin (0.25 g/kg via intraperitoneal injection) and perfused with PBS and neutrally buffered in 4% paraformaldehyde as previously described [14]. Brains were removed, postfixed over 6 h, and sectioned at a thickness of 40 μm. The sections were incubated with the following antibodies for 1–2 days at 4 °C: anti-Aβ_1–16_ (6E10) (#803015, Biolegend, San Diego, CA, USA, 1:1000), anti-iNOS (sc-7271, Santa Cruz Biotechnology, Dallas, TX, USA, 1:20), and anti-ionized calcium-binding adapter molecule 1 (IBA-1) (ab5076, Abcam, Cambridge, UK, 1:200). The sections were washed and then incubated with Alexa Fluor 594- and/or 488-conjugated secondary antibodies (Invitrogen, 1:1000). The binding specificity of the primary antibodies was confirmed with secondary antibody-only controls. Digital images were obtained using a confocal microscope equipped with an Olympus Disk Spinning Unit (Olympus, Tokyo, Japan). Fluorescence intensity or signal-positive cell numbers were analyzed using ImageJ software (NIH, RRID:SCR_003070).

### 2.7. Statistical Analysis

Data were summarized and statistically analyzed using GraphPad Prism 7 (GraphPad Software, RRID:SCR_002798). All data are presented as means ± the standard error of the means (SEM). Statistical significance (*p* < 0.05) was determined using 1-way or 2-way ANOVA with Tukey’s post hoc tests. Detailed statistical analysis methods are provided in the figure legends.

## 3. Results

### 3.1. Gln Supplementation Maintained Glutamatergic Neurotransmission Activity in the IL

**Figure 1:**
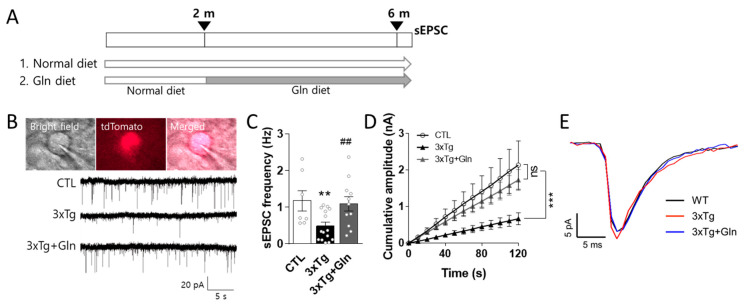
Glutamine (Gln) supplementation protected glutamatergic neurotransmission activity in 3×Tg-AD mice. (**A**) Dietary groups and experimental schedule. (**B**) Microscopic images of glutamatergic neurons in the infralimbic cortex utilized for measuring spontaneous excitatory postsynaptic current (sEPSC) and representative sEPSC signals. (**C**) sEPSC frequency. (**D**) Cumulative amplitude. Vglut2-ires-Cre::tdTomato (CTL; *n* = 7 cells; 2 mice), 3×Tg-Vglut2-ires-Cre::tdTomato (3×Tg; *n* = 13 cells; 3 mice), and Gln-supplemented 3×Tg (3×Tg+Gln; *n* = 11 cells; 3 mice). (**E**) Representative superimposed sEPSC traces. More than 30 peaks in each group were superimposed. Bars represent the means ± SEM. ** *p* < 0.01 vs. CTL, and ## *p* < 0.01 vs. 3×Tg in 1-way ANOVA with Tukey’s multiple comparison tests. *** *p* < 0.001 in 2-way ANOVA with Tukey’s multiple comparison tests.

To evaluate the effects of Gln on glutamatergic neurotransmission in 3×Tg-AD mice, female Vglut2-ires-Cre::tdTomato or 3×Tg-Vglut2-ires-Cre::tdTomato mice were fed a normal or Gln-supplemented diet beginning at 2 months of age. sEPSCs were analyzed in the IL at 6 months of age (Figure 1A): CTL (control; normal diet Vglut2-ires-Cre::tdTomato group; *n* = 7 cells; 2 mice), 3×Tg (normal diet 3×Tg-Vglut2-ires-Cre::tdTomato group; *n* = 13 cells; 3 mice), and 3×Tg+Gln (Gln-supplemented 3×Tg group; *n* = 11 cells; 3 mice) (Figure 1A).

sEPSCs were measured in the fluorescently labeled glutamatergic neurons in the IL (Figure 1B). The sEPSC frequency in the IL was significantly decreased in the 3×Tg group (CTL vs. 3×Tg; *p* = 0.0097). The cumulative amplitude in the 3×Tg group was obviously lower than that in the CTL group (Figure 1C,D), although the sEPSC amplitude in the 3×Tg group did not change (Figure 1E). However, the sEPSC frequency and cumulative amplitude in the 3×Tg+Gln group were significantly higher than that in the 3×Tg group and comparable to that in the CTL group (Figure 1B–D; *p* = 0.0075 in 3×Tg vs. 3×Tg+Gln; *p* = 0.8082 in CTL vs. 3×Tg+Gln). There were no differences in the current rise and decay time between groups (Figure 1E).

### 3.2. Gln Supplementation Prevented MCI Onset in 3×Tg Mice

**Figure 2:**
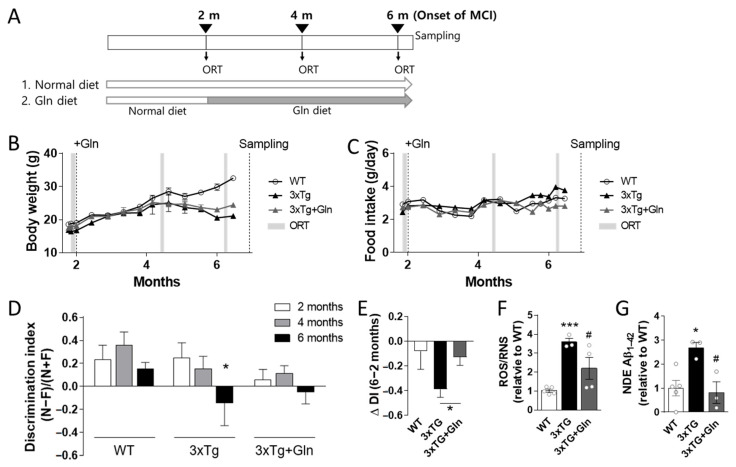
Gln supplementation in 6-month-old 3×Tg-AD mice exhibited inhibitory effects on mild cognitive impairment (MCI) and the induction of oxidative stress and amyloid beta (Aβ) accumulation in the plasma. (**A**) Dietary groups and experimental schedule. (**B**) Body weights and (**C**) food intake were measured weekly. (**D**) Discrimination index (DI) in the object recognition test (ORT) in wild-type (WT, *n* = 5), 3×Tg-AD (3×Tg, *n* = 3), and Gln-diet 3×Tg (3×Tg+Gln, *n* = 4) mice at 2, 4, and 6 months of age. (**E**) Change in DI values in the ORT in mice between the age of 2 and 6 months. (**F**) Reactive oxygen/nitrogen species (ROS/RNS) analysis in plasma. (**G**) Aβ_1–42_ content in neuron-derived exosomes (NDEs) isolated from plasma. ELISA results of Aβ_1–42_ were normalized to those for CD81. Bars represent the means ± SEM. * *p* < 0.05, *** *p* < 0.001 vs. WT, and # *p* < 0.05 vs. 3×Tg in 1-way ANOVA with Tukey’s multiple comparison tests.

Cognitive function was examined in 2-month-old female 3×Tg and WT mice. The mice were then randomly separated into three groups: WT (normal diet WT group; *n* = 5), 3×Tg (normal diet 3×Tg-AD; *n* = 3), and 3×Tg+Gln (Gln-supplemented 3×Tg, *n* = 4) (Figure 2A). Body weights in the 3×Tg and 3×Tg+Gln groups were lower than those in the WT group, and food intake did not differ between groups. There were no differences in body weight and food intake between the 3×Tg and 3×Tg+Gln groups, indicating that Gln supplementation did not affect body weight or food intake (Figure 2B,C).

Cognitive function was examined every 2 months until the 3×Tg group showed MCI. Object recognition memory in the ORT was worse at 6 months than at 2 months in the 3×Tg group (2 vs. 6 months; *p* = 0.0265 in paired *t*-test). However, this change was not significant in the 3×Tg+Gln group (Figure 2D; 2 vs. 6 months; *p* = 0.1563 in paired *t*-test). The change in the DI between 2 and 6 months (ΔDI_6-2 months_) in the 3×Tg group was more significant than that in the 3×Tg+Gln group (Figure 2D,E; *p* = 0.0425; 3×Tg vs. 3×Tg+Gln). Additionally, a significant increase in ROS/RNS levels was found in the plasma of the 3×Tg group compared with the 3×Tg+Gln group (Figure 2F; *p* < 0.0001 in WT vs. 3×Tg; *p* = 0.0461 in 3×Tg vs. 3×Tg+Gln). Exosomal Aβ_1–42_ levels in the plasma, which is a recently suggested non-invasive biomarker for MCI and AD [26,27,28], also increased in the 3×Tg group but not in the 3×Tg+Gln group (Figure 2G; *p* = 0.0107 in WT vs. 3×Tg; *p* = 0.0213 in 3×Tg vs. 3×Tg+Gln).

### 3.3. Gln Prevented Increases in Amyloid, iNOS, and IBA-1 Contents in 3×Tg Mice

**Figure 3:**
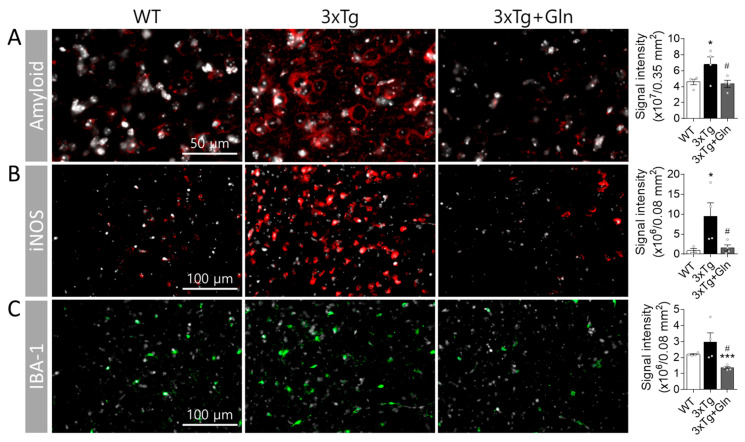
Gln supplementation prevented increases in amyloid content and expression of proteins related to ROS/RNS production in the infralimbic cortex of 6-month-old 3×Tg mice. Representative immunohistochemistry images and signal intensity quantification for amyloid (**A**), IBA-1 (**B**), and iNOS (**C**). The white signal corresponds to DAPI. Red or green signals represent target proteins. Scale bar sizes are indicated in the images. Bars represent the means ± SEM. *n* = 4/group. * *p* < 0.05, *** *p* < 0.001 vs. WT, and # *p* < 0.05 vs. 3×Tg in 1-way ANOVA with Tukey’s multiple comparison tests.

To investigate the activation of glutamatergic neurotransmission by Gln and its preventative effect against MCI in 3×Tg mice, we analyzed the contents of amyloid, iNOS, and IBA-1 in the PFC (*n* = 4/group). The intracellular amyloid precursor protein (APP), immunized with the 6E10 antibody, showed a greater increase in the IL of the 3×Tg group compared with the WT group at 6 months (*p* = 0.0387; WT vs. 3×Tg). However, Gln supplementation prevented the induction of amyloid in the IL (Figure 3A; *p* = 0.0313; 3×Tg vs. 3×Tg+Gln). At this age, extracellular Aβ plaques were not observed in 3×Tg mice.

The expression level of iNOS in the IL was 10 times higher in the 3×Tg group than in the WT group, with intense signals scattered throughout the frontal cortex. However, iNOS expression in the 3×Tg+Gln group was maintained at a similar level to that in the WT group (Figure 3B; *p* = 0.0451 in WT vs. 3×Tg; *p* = 0.0325 in 3×Tg vs. 3×Tg+Gln). IBA-1 expression in the IL did not show a significant change in the 3×Tg group. However, IBA-1 expression was lower in the 3×Tg+Gln group compared with the normal diet groups (Figure 3C; *p* = 0.1257 in WT vs. 3×Tg; *p* < 0.0001 in WT vs. 3×Tg+Gln; *p* = 0.0183 in 3×Tg vs. 3×Tg+Gln). At 6 months, there were no significant changes in the expression levels of APP, iNOS, and IBA-1 in the hippocampus of the 3×Tg group mice (Supplementary Figure S1).

## 4. Discussion

In this study, Gln supplementation maintained glutamatergic neuronal activity in the IL of 3×Tg-AD mice at the typical age of MCI onset. At 6 months, the sEPSC frequency in the 3×Tg+Gln group was higher compared with the 3×Tg group and similar to that in the WT group. As there were no differences in the rise and decay times of sEPSCs between the groups, changes in glutamatergic activity in the 3×Tg and 3×Tg+Gln groups were primarily attributed to alterations in sEPSC frequency rather than the characteristics of sEPSC peaks.

It is widely known that hypoactive glutamatergic neurotransmission is a major contributor to cognitive disorders [2,3]. Therefore, we evaluated whether Gln supplementation affects the cognitive impairment observed in 3×Tg-AD mice. Remarkably, the cognitive function of the 3×Tg+Gln group was not significantly decreased at 6 months of age, while the 3×Tg group exhibited signs of MCI. Moreover, the rate of cognitive decline (∆DI_6-2 months_) in the 3×Tg+Gln group remained similar to the normal cognitive decline associated with aging in the WT group. This provides evidence that Gln supplementation can suppress MCI in a genetic dementia model, as well as in a CIS-induced depression and MCI model.

In our experiments, Gln was administered to mice through rodent chow containing 150 mg Gln/kg chow, which corresponded to approximately 450 μg Gln/day/25 g mouse considering the food intake data. This is equivalent to approximately 90 mg Gln/day/60 kg human, taking into account the differences in body surface area [29]. Importantly, during the 2–6-month period of Gln supplementation, there were no significant differences in body weight and food intake between the 3×Tg+Gln and 3×Tg groups. It should be noted that this Gln dosage was not sufficient to completely reverse the hypermetabolic state and restore normal body weight in the 3Tg-AD mice, as observed in Figure 2 and previous reports [30,31]. However, despite this limitation, Gln demonstrated significant cognitive protective effects at this dosage. These results suggest that Gln can prevent or at least delay cognitive decline in 3×Tg-AD mice without imposing a metabolic burden or exhibiting toxicity.

Considering the results of previous studies, the effects of Gln supplementation on glutamatergic neuronal activity and cognitive functions can be explained via two mechanisms: (1) protective effects on Glu-Gln levels and the Glu-Gln cycle [6,9,12], and (2) inhibitory effects on oxidative/nitrative stress and inflammation [14]. However, changes in Glu-Gln levels in the brain were not observed in 7-month-old 3×Tg-AD mice [31]. Furthermore, a decrease in GS expression levels in the brains of 3×Tg-AD mice is typically observed after 9 months of age and is clearly observed only after 12 months of age [32]. In the present study, we also did not observe changes in the Glu-Gln levels or Glu-Gln transporter expression in the PFC at 6 months, even with Gln supplementation in the 3×Tg-AD mice (Supplementary Figure S2A–D). Similarly, GS expression levels and activity were not altered in either the 3×Tg or 3×Tg+Gln groups (Supplementary Figure S2E,F). Therefore, Glu-Gln levels and GS activity may not significantly influence the onset of MCI, at least in 6-month-old 3×Tg mice. As a result, in this study, we focused on analyzing the protective effects of Gln supplementation on cognitive function, specifically examining its antioxidant and anti-inflammatory activities. Gln supplementation effectively inhibited oxidative stress and inflammation-related changes, such as amyloid accumulation, microglia increase, and iNOS induction, in 3×Tg-AD mice.

The Gln diet effectively reduced amyloid deposits both in the PFC and plasma NDEs in 3×Tg-AD mice. Aβ deposition leads to oxidative stress and cognitive impairment [23,24]. In 3×Tg-AD mice, APP or Aβ deposits in the frontal cortex are associated with the onset of MCI at 4–6 months of age [20,21,22]. Recent research suggests that not only extracellular Aβ plaques but also intracellular deposits of Aβ and APP may play a role in the pathogenesis of MCI and AD [20,33]. In our study, we observed an increase in intracellular amyloid expression in the IL of 6-month-old 3×Tg mice, despite the absence of extracellular Aβ plaques. Although it remains unclear whether Gln-supplementation inhibited amyloid expression/accumulation or facilitated its degradation, we observed minimal amyloid-positive signals in the IL of 3×Tg+Gln group, which is consistent with the low levels of NDE Aβ_1–42_ in the plasma.

Accumulating studies have provided evidence for the utility of Aβ_1–42_ and phospho-tau levels in plasma NDEs as non-invasive biomarkers for predicting the development of MCI and AD [27,34]. Additionally, several NDE biomarkers, including Aβ_1–42_, have shown promise in predicting the transition from MCI to AD dementia [27,35,36,37]. In MCI patients, NDE concentrations of Aβ_1–42_ and phospho-tau are lower in MCI patients compared with patients with ADC (MCI converting to AD) and AD [27]. In our study, we observed a significant increase in plasma NDE Aβ_1–42_ levels in the 3×Tg group compared with the CTL group, confirming the presence of at least the MCI state in the 6-month-old 3×Tg mice. However, Gln supplementation mitigated MCI advancement, once again highlighting the inhibitory effect of Gln on amyloid accumulation.

The expression of APP is highly prevalent at synapses [38], and as a result, amyloid accumulation and the ensuing oxidative stress have widespread effects on neurons and glial cells at the synapses, leading to decreased neuronal activity [39,40]. The reduced amyloid protein accumulation observed in the IL of the 3×Tg+Gln group is likely the primary reason for the protective effect of Gln on glutamatergic neuronal activity. Further research is warranted to elucidate the intricate mechanisms underlying how Gln prevents amyloid deposits.

One possibility is that Gln may inhibit amyloid accumulation through its antioxidant properties. In this study, Gln supplementation resulted in decreased ROS/RNS plasma levels and reduced expression of oxidative stress/inflammation-related proteins, including iNOS and IBA-1, in 3×Tg-AD mice. iNOS induces nitric oxide production in an oxidative environment, promoting peroxynitrite formation. iNOS also induces the microglia inflammatory responses and expression of inflammatory factors, such as IL-6 [41]. IBA-1 is a microglia/macrophage cytoskeletal protein that supports phagocytosis. Microglia are activated and proliferate rapidly in response to exogenous and endogenous stressors, including oxidative stress [42]. Microglia/macrophage numbers and inflammation-related protein expression are increased in the brains of 6-month-old 3×Tg-AD mice [42]. These alterations were also observed in the present study; however, Gln supplementation suppressed these changes in the IL of 3×Tg-AD mice.

Recent studies provide compelling evidence indicating a reciprocal relationship between amyloid expression/oligomerization and oxidative stress. Oxidative stress induces APP and Aβ production. Conversely, APP and its non-amyloidogenic fragments can enhance mitochondrial metabolism and ROS production [39,40]. Aβ oligomers and APP can interact with redox-active metal ions, such as copper, iron, and zinc. Their binding promotes Aβ oligomerization, resulting in the oxidative damage of proteins and lipids, including Aβ itself [23,43,44]. Additionally, Aβ deposition activates microglia, which are a major ROS source in the brain. Although microglia initially respond to and counteract Aβ deposition, high levels of accumulated Aβ can sustain microglia activation, triggering chronic inflammation and ROS/RNS overproduction that contribute to oxidative/nitrative stress in AD [42]. Therefore, Gln, with its antioxidant properties, may interrupt the detrimental cycle of oxidative stress and amyloid accumulation, ultimately slowing down the progression of MCI.

While Gln is typically considered a non-essential amino acid under normal conditions, it becomes a conditionally essential amino acid in hazardous environments, such as stress and injury, playing a vital role in maintaining redox homeostasis. Gln is involved in the synthesis of the antioxidant glutathione, which facilitates the detoxification of both xenobiotic and endogenous substances [45,46]. Additionally, Gln has the potential to suppress apoptosis triggered by oxidative/nitrative stress [45,47]. Thus, Gln has been employed in clinical settings to safeguard tissues against damage caused by ROS/RNS [45,48]. These results support the hypothesis that the cognitive and glutamatergic neuroprotective effects of Gln observed in this study may be attributed to its antioxidative properties.

Further investigation is necessary to gain a clear understanding of the underlying mechanism behind the protective effect of Gln on cognitive function. Specifically, establishing the causal relationship between the inhibitory effect of Gln supplementation on amyloid accumulation and its antioxidant effects, as well as identifying the limitations of its efficacy, would be beneficial for utilizing Gln as a potential treatment for MCI. However, the present study demonstrated that Gln supplementation can protect glutamatergic neurotransmission and delay the onset of MCI in 3×Tg-AD mice, which is consistent with findings from previous studies. These results highlight the potential of a Gln-supplemented diet in delaying MCI onset, not only in a model of MCI induced by chronic stress but also in a mouse model predisposed to cognitive impairment and dementia. Therefore, Gln holds promise as a cognitive function protective agent. Furthermore, this study is the first, to the best of our knowledge, to report the inhibitory effects of Gln supplementation on amyloid accumulation in the PFC and the onset of MCI in 3×Tg-AD mice.

## 5. Conclusions

This study provides evidence that Gln can act as a nutraceutical agent, offering protection to glutamatergic activity in the PFC against oxidative damage induced by amyloid protein accumulation. Ultimately, this protective effect of Gln contributes to delaying the onset of MCI.

## Data Availability

Not applicable.

## Supplementary Materials

The following supporting information can be downloaded at: https://www.mdpi.com/article/10.3390/nu15122794/s1, Supplementary Figure S1. There were no significant changes in the expression levels of APP, iNOS, and IBA-1 in the hippocampus of 6-month-old 3×Tg-AD mice. Signal intensity quantification in immunohistochemical analysis for amyloid (A), IBA-1 (B), and iNOS (C). WT (normal diet wild-type group), 3×Tg (normal diet 3×Tg-AD), and 3×Tg+Gln (Gln-supplemented 3×Tg). *n* = 4/group. Bars represent the means ± SEM. * *p* < 0.05 vs. WT and # *p* < 0.05 vs. 3×Tg in 1-way ANOVA with Tukey’s multiple comparison tests. Supplementary Figure S2. There were no significant changes in Glu-Gln levels (A,B), Glu-Gln transporter expression (C,D), GS expression (E), and GS activity (F) in the prefrontal cortex of 6-month-old 3×Tg-AD mice. WT (normal diet WT group; *n* = 5), 3×Tg (normal diet 3×Tg-AD; *n* = 3), and 3×Tg+Gln (Gln-supplemented 3×Tg, *n* = 4). Bars represent the means ± SEM. 1-way ANOVA with Tukey’s multiple comparison tests.
