# Supplementary material for: Glutamine Supplementation Preserves Glutamatergic Neuronal Activity in the Infralimbic Cortex, Which Delays the Onset of Mild Cognitive Impairment in 3×Tg-AD Female Mice
Source: Nutrients. 2023 Jun 19;15(12):2794. doi: 10.3390/nu15122794 (PMC10303714; doi:10.3390/nu15122794)
Supplement: Supplementary file 1 [file nutrients-15-02794-s001.zip › nutrients-2444433-supplementary.pdf]

Supplementary Figure S1.

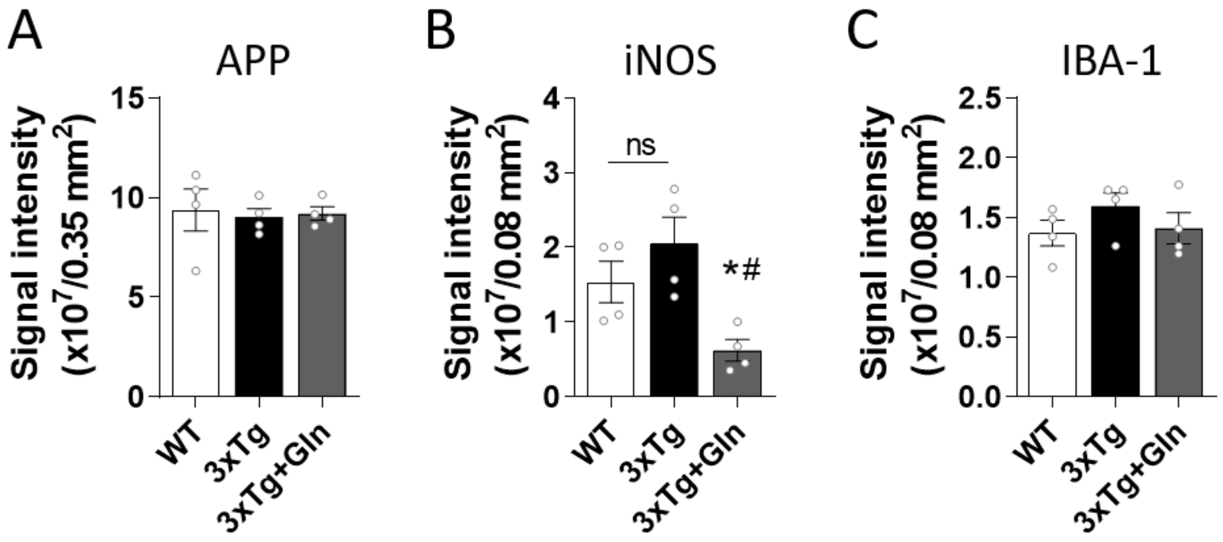

Supplementary Figure S1. There were no significant changes in the expression levels of APP, iNOS, and IBA-1 in the hippocampus of 6-month-old 3×Tg-AD mice. Signal intensity quantification in immunohistochemical analysis for amyloid (**A**), IBA-1 (**B**), and iNOS (**C**). WT (normal diet wild-type group), 3×Tg (normal diet 3×Tg-AD), and 3×Tg+Gln (Gln-supplemented 3×Tg). $n = 4$/group. Bars represent the means ± SEM. * $p < 0.05$ vs. WT and # $p < 0.05$ vs. 3×Tg in 1-way ANOVA with Tukey's multiple comparison tests.

Supplementary Figure S2.

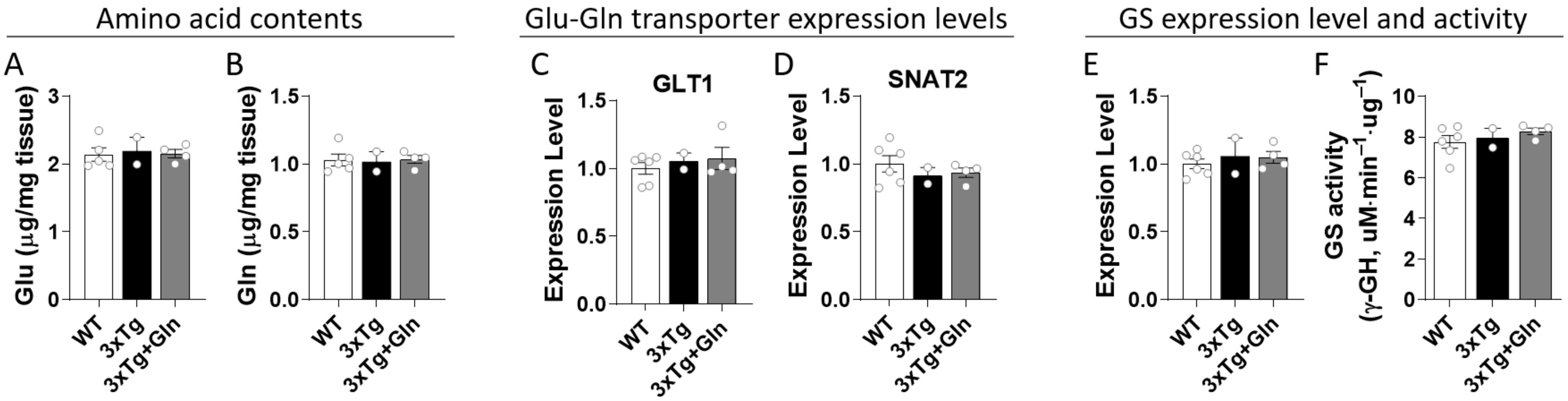

Supplementary Figure S2. There were no significant changes in Glu-Gln levels (**A,B**), Glu-Gln transporter expression (**C,D**), GS expression (**E**), and GS activity (**F**) in the prefrontal cortex of 6-month-old 3×Tg-AD mice. WT (normal diet WT group; n = 5), 3×Tg (normal diet 3×Tg-AD; n = 3), and 3×Tg+Gln (Gln-supplemented 3×Tg, n = 4). Bars represent the means ± SEM. 1-way ANOVA with Tukey's multiple comparison tests.
